# Efficacy and mechanisms of behavioral therapy components for insomnia coexisting with chronic obstructive pulmonary disease: study protocol for a randomized controlled trial

**DOI:** 10.1186/s13063-016-1334-0

**Published:** 2016-05-23

**Authors:** Mary C. Kapella, James J. Herdegen, Franco Laghi, Alana D. Steffen, David W. Carley

**Affiliations:** Department of Biobehavioral Health Science, College of Nursing, University of Illinois at Chicago, Chicago, USA; Sleep Medicine, Rush University Medical Center, Chicago, IL USA; Edward Hines, Jr. Department of Veterans Affairs Hospital, Hines, IL USA; Division of Pulmonary and Critical Care, Medicine, Loyola University Stritch School of Medicine, Maywood, IL USA; UIC Center for Narcolepsy, Sleep and Health Research, Chicago, USA; Department of Health Systems Science, College of Nursing, University of Illinois at Chicago, Chicago, IL USA

**Keywords:** Chronic obstructive pulmonary disease, Insomnia, Cognitive behavioral therapy

## Abstract

**Background:**

Difficulty falling asleep, staying asleep or poor-quality sleep (insomnia) is common in people with chronic obstructive pulmonary disease (COPD). Insomnia is related to greater mortality and morbidity, with four times the risk of mortality for sleep times below 300 min. However, insomnia medications are used with caution in COPD due to their potential adverse effects. While cognitive behavioral therapy for insomnia (CBT-I) is effective for people with primary insomnia and people with other chronic illnesses, the efficacy and mechanisms of action of such a therapy are yet unclear in people with both insomnia and COPD. The purpose of this study is to rigorously test the efficacy of two components of insomnia therapy – CBT-I and COPD education (COPD-ED) – in people with coexisting insomnia and COPD, and to identify mechanisms responsible for therapy outcomes. The rationale for the proposed study is that once the efficacy and mechanisms of CBT-I and COPD-ED are known, new and innovative approaches for insomnia coexisting with COPD can be developed to non-pharmacologically minimize insomnia and fatigue, thereby leading to longer, higher-quality and more productive lives for people with COPD, and reduced societal cost due to the effects of insomnia.

**Methods:**

We are conducting a randomized, controlled, parallel-group (*N* = 35 each group) comparison of CBT-I, COPD-ED and non-COPD, non-sleep health education Attention Control (AC) using a highly efficient four-group design. Arm 1 comprises 6 weekly sessions of CBT-I + AC; Arm 2 = 6 weekly sessions of COPD-ED + AC; Arm 3 = 6 weekly sessions of CBT-I + COPD-ED; and Arm 4 = 6 weekly sessions of AC. This design will allow completion of the following specific aims: (1) to determine the efficacy of individual treatment components, CBT-I and COPD-ED, on insomnia and fatigue, (2) to define the mechanistic contributors to the outcomes after CBT-I and COPD-ED.

**Discussion:**

The research is innovative because it represents a new and substantive departure from the usual insomnia therapy, namely by testing traditional CBT-I with education to enhance outcomes. The work proposed in aims 1 and 2 will provide systematic evidence of the efficacy and mechanisms of components of a novel approach to insomnia comorbid with COPD. Such results are highly likely to provide new approaches for preventive and therapeutic interventions for insomnia and fatigue in COPD.

**Trial registration:**

ClinicalTrials.gov Identifier: NCT01973647. Registered on 22 October 2013.

## Background

Approximately one fourth of adults worldwide have chronic obstructive pulmonary disease (COPD) [1], and half of them suffer from insomnia [2] (difficulty falling or staying asleep, or poor-quality sleep that interferes with daytime functioning [3]). Insomnia is related to greater mortality [4–6], with studies showing *four times the mortality risk* for sleep times below 300 min [5, 6]. Insomnia also produces morbidity – insomnia sufferers generate 75 % greater healthcare costs than individuals without insomnia [7]. Lost productivity from insomnia is estimated at US$63.2 billion annually in the US alone [8]. People with COPD experience debilitating fatigue and a gradual decline in function that are partly related to insomnia [9–11]. However, insomnia medications are used with caution in COPD due to potential respiratory effects, hypoxia and effects on cognition [12]. Common features of COPD, such as dyspnea, chronic inflammation and emotional arousal (anxiety and depression), also affect insomnia and can interfere with therapy outcomes. Cognitive behavioral therapy for insomnia (CBT-I), a therapy that provides guidance on changing unhelpful sleep-related beliefs and behavior, is a promising non-pharmacological treatment that has demonstrated effectiveness for insomnia, but the efficacy of components and mechanisms of action of CBT-I have not been thoroughly examined in people with insomnia coexisting with COPD.

Insomnia is prevalent in people with medical disorders, and therapy for insomnia might improve these medical disorders [13]. However, there are only a limited number of randomized, controlled studies in this area. Although evidence obtained from studies that included participants with cancer [14, 15], renal failure [16], chronic pain [17], osteoarthritis, [18] and COPD [19, 20] support the notion that CBT-I is effective in reducing insomnia when coexisting with chronic illness, no studies to date have examined CBT-I specifically for people with COPD. It is yet undetermined whether improving self-efficacy for the medical illness (i.e., COPD) mediates outcomes after CBT-I, but our preliminary data suggest that education on COPD topics, such as management of dyspnea and COPD exacerbations, may improve self-efficacy and reduce depression, indirectly reducing insomnia and fatigue [20].

Our long-term goal is to help develop safe and effective non-pharmacological interventions to minimize insomnia and its consequences in people with COPD. The purpose of this study is to systematically test the efficacy of two components of therapy for people with coexisting insomnia and COPD – CBT-I and COPD education (COPD-ED) – and to identify the mechanisms responsible for therapeutic outcomes. Our central hypothesis is that both CBT-I and COPD-ED will have positive, lasting effects on objectively and subjectively measured insomnia and fatigue. This hypothesis is consistent with preliminary data from our pilot study [20] comparing CBT-I and COPD-ED in people with insomnia coexisting with COPD. The pilot study results demonstrated feasibility of the two components and provided preliminary evidence of positive effects on outcomes, which differed in men and women. Given the prevalence of insomnia and its consequences, it is important not only to identify the most effective approach to minimize insomnia for those with COPD but also to identify the mechanisms responsible for the outcomes, as not all patients will benefit from CBT-I. The rationale for this study is that once the efficacy and mechanisms of CBT-I and COPD-ED are known, new and innovatively tailored interventions can be developed to non-pharmacologically minimize insomnia and fatigue, thereby leading to longer, higher-quality and more productive lives for people with COPD, and reduced societal cost due to the effects of insomnia. The clinical gains could be, unlike those of pharmacotherapy, sustained following treatment discontinuation. We are testing our central hypothesis by completing a randomized, parallel-group, 2 × 2 factorial design which results in four groups (*N* = 35 each group): CBT-I, COPD-ED, both CBT-I and COPD-ED, and neither [21, 22].

Results from previous studies of predictors of positive outcomes of CBT-I suggest that mechanisms include changes in sleep-related beliefs, sleep habits, emotional arousal and self-efficacy for sleep [23]. Thus, our contribution here is expected to be knowledge of the efficacy and mechanisms of the components of a novel insomnia therapy for people with COPD and a detailed understanding of which patients are most likely to benefit from the therapy. This contribution will be significant because it is a necessary step in the development of effective non-pharmacological therapies for insomnia coexisting with COPD. Once such advances in therapy for insomnia coexisting with COPD become available, results can be used to further develop and make available effective and efficient insomnia therapies for people with COPD. Components of therapy found to be most important for positive outcomes can be included in new, more efficient therapies. It is expected that what we learn will be equally applicable to the prevention of insomnia and fatigue in people with COPD, potentially leading to them having longer, higher-quality and more productive lives and to society having to burden lower costs related to insomnia in COPD.

We are testing components of a novel therapy (CBT-I and COPD-ED) in an understudied population using a highly efficient study design. Despite the established need for such a therapy for people with COPD, this group is understudied, perhaps because of challenges such as recruiting subjects with COPD and managing periodic exacerbations of COPD that could occur during treatment. Furthermore, people with insomnia coexisting with COPD are subject to exacerbations of their illness that predispose them to recurrent insomnia and may interfere with CBT-I outcomes. The research represents a new and substantive departure from the usual insomnia therapy, specifically by combining traditional CBT-I with disease-specific education (COPD-ED) to enhance self-efficacy for the management of COPD. Enhancing self-efficacy for COPD is likely to reduce insomnia and fatigue by attenuating the anxiety and depressed mood associated with the disease.

### Aim 1

To determine the efficacy of individual treatment components, CBT-I and COPD-ED, on insomnia and fatigue. Our hypothesis is that both components will decrease insomnia and fatigue at the end of the six-session treatment period, and that these differential effects will be sustained for at least 3 months post treatment.

### Aim 2

To define the mechanistic contributors to the outcomes after CBT-I and COPD-ED. Our hypothesis is that CBT-I and COPD-ED components impact insomnia and fatigue through complementary mechanisms. Positive changes in beliefs about sleep, sleep habits, self-efficacy for management of COPD (SEC) and sleep (SES), and reduced emotional arousal (EA) will mediate the improving conditions in insomnia and fatigue and that gender, inflammation and functional status will moderate the outcomes.

## Methods: participants, interventions and outcomes

### Conceptual framework

**Fig. 1:**
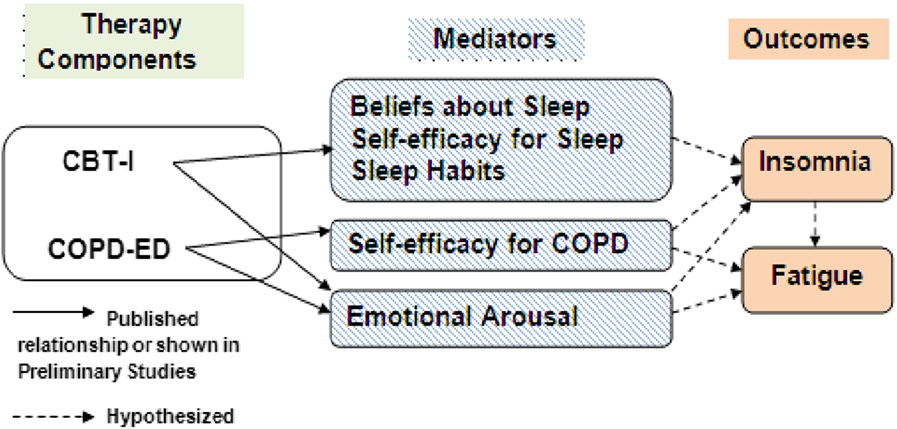
Conceptual model

The conceptual framework (Fig. 1) for this proposal has its foundations in social learning theory [24] and cognitive behavioral theory [25], which emphasize the interplay of cognitive, behavioral and environmental factors. According to these theories, in order to participate in behavioral change, persons must possess the knowledge, skills and confidence necessary to self-regulate behavior and have a firm belief in their ability to initiate change and change these behaviors. All individuals have personal traits that influence their predisposition to insomnia by impacting the degree to which they are physiologically, emotionally or cognitively hyper-aroused and unable to sleep. In our conceptual framework, COPD-related factors contributing to the illness burden can be predisposing or precipitating factors for insomnia. Cognitive factors, such as dysfunctional beliefs about sleep, emotional arousal such as anxiety and depression, and low self-efficacy for sleep and self-management of COPD, can also contribute [26]. For people with COPD, insomnia is likely to increase symptom burden and affect the ability to function in daily life. The components of our intervention target the factors that contribute to insomnia and promote its chronicity [27]. The efficacy of CBT-I on outcomes in COPD is unclear but must be clarified to develop effective non-pharmacological interventions specifically for insomnia coexisting with COPD. Future CBT-I programs will benefit from this information, which will provide guidance on potential components to include in CBT-I.

### Trial design

We are conducting a 2 × 2 factorial randomized trial where participants are randomized into four groups created by crossing the two factors, CBT-I versus Attention Control (AC)1 and COPD-ED versus AC2 (*N* = 35 each group). Arm 1 is comprised of 6 weekly sessions of CBT-I + AC1; Arm 2 = 6 weekly sessions of COPD-ED + AC2; Arm 3 = 6 weekly sessions of CBT-I + COPD-ED; and Arm 4 = 6 weekly sessions of AC1 + AC2. Each session is approximately 75 min in length. There is follow-up at 3 months post intervention. Dependent variables include objectively and subjectively measured insomnia and fatigue, measured three times: at baseline (pretest, T1), immediately post intervention (posttest, T2), and at 3 months (follow-up, T3) to provide data on the long-term effects of the interventions. In addition to these times, mediator variables are measured after the fourth session to improve our ability to test a causal mediating effect.

#### Setting and sample

The setting for the study is the University of Illinois at Chicago (UIC) College of Nursing Center for Narcolepsy, Sleep and Health Research (CNSHR) and Edward Hines, Jr. Veterans Administration (VA) Medical Center. Institutional Review Board approval was obtained from both institutions and all subjects sign a written informed consent. A total of 166 subjects with mild to very severe COPD as defined by the GOLD standards will be randomly assigned to one of the four groups. Subjects will be stratified by disease severity and gender.

*Inclusion criteria* include: (1) mild to very severe COPD. Severity of COPD is defined according to the new GOLD standards [28], (2) age 45 years or older with no other major health problems, (3) clinical stability at the time of enrollment into the study without major exacerbation of COPD within the previous 2 months, (4) difficulty initiating or maintaining sleep, waking up too early or poor-quality sleep (insomnia) [3].

*Exclusion criteria* include: (1) evidence of restrictive lung disease or asthma, (2) pulse oximetry (oxygen saturation: SaO_2_) reading of less than 90 % at rest or less than 85 % at night for more than 5 min, (3) evidence of a major sleep disorder other than insomnia, (4) hypnotic use, (5) acute respiratory infection within the previous 2 months, (6) the presence of a potentially debilitating disease such as cancer, congestive heart failure, kidney disease, liver failure or cirrhosis; evidence of alcohol or drug abuse, musculoskeletal or degenerative nerve disease. (7) a self-reported current diagnosis of major depression or psychiatric disease or a Hospital Anxiety and Depression Scale (HADS) depression score of >11, (8) currently participating in pulmonary rehabilitation. Potential subjects are screened to ensure that they meet the inclusion criteria.

#### CBT-I, COPD-ED and AC interventions

For both groups, sessions consist of six weekly group sessions, 75 min in length, administered in an individual or small-group (two to four persons) setting. The sessions are provided by clinicians trained in CBT-I, COPD-ED and the AC health education. Although the intervention period is relatively brief, we expect that some subjects will miss sessions. Subjects are allowed to make up classes within 2 weeks, for up to two sessions. The CBT-I component is based on Morin [29] and Perlis [30] and includes a program of stimulus control therapy (SCT), sleep-restriction therapy, sleep hygiene, cognitive therapy and relaxation therapy. The COPD education component (COPD-ED) is modeled after the COPD education program used in a previous study. Briefly, topics include how the lungs work; overview of COPD; managing COPD, including techniques for managing dyspnea; discussion of COPD medications; preventing and coping with exacerbations, flu and colds; and preventing complications of COPD. Table 1 summarizes the components of the CBT-I and COPD-ED interventions. These topics are presented using brief PowerPoint presentations, discussion and handouts. The CBT-I + COPD-ED intervention is a combination of CBT-I and COPD education. Each intervention session contains a CBT-I segment and a COPD education segment, for a total of approximately 75 min (Table 1). Topics include managing insomnia and COPD using strategies known to be effective. The AC intervention is comprised of six sessions of social support and general health education. Education excludes sleep and COPD topics and includes information about topics such as cholesterol, blood pressure, color perception, vitamins and memory.

**Table 1:** CBT-I and COPD-ED topics

Session	CBT-I	COPD-ED
1	Orientation, “Background on sleep”	Orientation, “How the lungs work”
2	What is insomnia?	What is COPD?
3	Managing insomnia	Managing COPD
4	Managing insomnia, medications	Managing COPD, medications
5	Managing insomnia, techniques	Managing COPD, exacerbations, flu, colds
6	Insomnia relapse prevention	Preventing complications of COPD

*CBT-I* cognitive behavioral therapy for insomnia, *COPD-ED COPD* education, *COPD* chronic obstructive pulmonary disease

#### Measures

Table 2 describes the variables, measures and frequency of measurement. A general description of each measure is provided below.

**Table 2:** Measures, frequency

	Screening	Pretest	4th session	Posttest	Follow-up
Blood draw	X			X	
Pulmonary function	X			X	
Sleep study	X				
Beliefs about sleep		X	X	X	X
Sleep habits		X	X	X	X
Self-efficacy for sleep		X	X	X	X
Self-efficacy for COPD		X	X	X	X
Mood		X	X	X	X
Insomnia	X	X		X	X
Fatigue		X		X	X
Health information	X				
Clinical assessment		Each session			
Daytime functioning		X		X	X
		X		X	X
Estimated time required	2.5 hrs	2.5 hrs	No additional visit time	3.0 hrs	2.0 hrs

*COPD* chronic obstructive pulmonary disease

#### Moderators

*Inflammation:* we measure C-reactive protein, commonly used as a marker of inflammation. C-reactive protein has been shown to be a valid marker of inflammation in people with COPD [31]. *Functional status:* pulmonary function tests (PFTs) as described in the screening procedures are performed to measure pulmonary functional status at baseline and at posttest.

#### Mediators

*Beliefs about sleep:* the Dysfunctional Beliefs and Attitudes about Sleep Scale (DBAS-30) [29] is used to measure sleep-related beliefs in subjects with insomnia. The scale consists of 30 items in five themes: misattributions or amplification of the consequences of insomnia, diminished perception of control and predictability of sleep, unrealistic sleep expectations, misconceptions about the causes of insomnia, and faulty beliefs about sleep-promoting practices. The modified response scale includes a 10-point Likert scale labeled *strongly disagree* to *strongly agree*. Adequate reliability and validity were demonstrated in older adults with and without sleep problems. The DBAS-30 was sensitive to changes in older adults with insomnia [32, 33]. *Self-efficacy for sleep:* We measure self-efficacy for sleep using the Self-Efficacy Scale for sleep (SES) [34], a nine-item questionnaire including a 5-point Likert scale. Respondents indicate their confidence (*not confident at all* to *very confident*) in falling asleep, staying asleep and obtaining refreshing sleep. Reliability, validity and test-retest reliability of the SES have been demonstrated in older adults [35–38]. *Sleep habits:* the sleep diary and actigraphy are used to measure sleep habits, including the timing of bedtime, naps, time spent in bed and strategies used when awake during the night. *Self-efficacy for COPD:* the COPD Self-efficacy Scale [39] (COPD SES) is used to measure self-efficacy for COPD. The COPD SES is a 34-item questionnaire with five subscales that includes a 5-point Likert scale. Respondents indicate their confidence in managing breathing (dyspnea) during intense emotions, physical exertion, adverse weather or environmental conditions and risk factors. Responses range from *very confident* to *not at all confident,* scoring 5 to 1 with 5 representing higher self-efficacy. Reliability and validity of the COPD SES have been demonstrated in people with COPD [40, 41].

#### Emotional arousal, fatigue and daytime functioning

To minimize response burden while simultaneously achieving high measurement precision, computerized adaptive testing (CAT) versions for fatigue, depression and anxiety (emotional arousal) and physical function from the Patient Reported Outcomes Measurement Information System (PROMIS) is used (www.nihpromis.org). The PROMIS item banks were developed by researchers from the NIH, using item response theory (IRT) for developing subjective measures with a high level of interval measurement precision, approaching the precision of physiological instruments. Participants respond to one item per tablet computer screen and selection of the next item is guided by the participant’s response to a previously administered item.

The PROMIS Fatigue item bank evaluates a range of mild subjective feelings of tiredness to an overwhelming, debilitating, and sustained sense of exhaustion. The depression item bank assesses self-reported negative mood, views of self, and social cognition, as well as decreased positive affect and engagement. Somatic symptoms (changes in appetite, sleeping patterns) are not included, which eliminates consideration of these items’ confounding effects when assessing patients with comorbid physical conditions. The anxiety item bank measures self-reported fear, anxious misery, hyper-arousal, and somatic symptoms related to arousal. The physical function item bank measures self-reported capability rather than actual performance of physical activities. CAT is appropriate for the adult general population and adults with chronic health conditions. CAT is generic rather than disease-specific and assesses the previous 7 days.

### Outcomes (dependent variables)

#### Insomnia

We objectively assess insomnia using the Actiwatch-2® (MiniMitter, Philips Respironics, Carlsbad, CA, USA) actigraph. Scoring of actigraph data with standardized computer algorithms is reliable and valid relative to polysomnography [42, 43]. Editing is done with sleep diary information because actigraphy tends to overestimate sleep during quiet nocturnal wakefulness, but is useful in measuring treatment effects [44]. The accelerometer is worn on the non-dominant wrist for a minimum of 4 days at each data collection. Insomnia variables measured include sleep onset latency (SL, minutes to fall asleep after lights out), wake after sleep onset (WASO, minutes spent wake after sleep onset), number of awakenings after sleep onset (NA), total sleep time (TST) and sleep efficiency (SE, time spent asleep divided by time in bed).

We subjectively measure insomnia severity using the Sleep Impairment Index (SII) [29, 45] and a sleep diary. The SII includes seven items, each rated on a 5-point scale (0 = not at all, 4 = extremely) to evaluate: sleep onset severity, sleep maintenance, early morning awakening problems, satisfaction with current sleep, interference with daily functioning, impairment attributed to the sleep problem, and level of distress caused by the sleep problem. Total scores range from 0 to 28, with high scores indicating greater insomnia severity. Validity and reliability were demonstrated in older people with insomnia [45]. A 6-point reduction is recommended to represent a clinically meaningful improvement in individuals with insomnia [46]. The sleep diary is completed by all CBT-I participants in the morning at breakfast time for a 1-week baseline period. Subjects receiving CBT-I complete sleep diaries daily during the six-session intervention period, for 1 week at the end of the intervention; and for 1 week at the 3-month follow-up. The sleep diary is completed by participants not receiving CBT-I for 1 week at baseline; 1 week at the end of the intervention; and 1 week at the 3-month follow-up. Sleep variables as above including SL, WASO, TST, EMA and SE are derived from the diary. Variables are calculated for each night and a weekly mean is computed. Sleep diaries have demonstrated validity and reliability in measuring insomnia in terms of sleep latency and waking after sleep onset [47, 48].

#### Fatigue

We measure the frequency and intensity of fatigue experienced in the previous 2 weeks using the Chronic Respiratory disease Questionnaire – Fatigue (CRQ-F) [49]. This consists of four items, scored on a 7-point Likert scale. Lower scores indicated more fatigue. The minimal clinically significant change in the CRQ score is associated with a change of 0.5, and a moderate change is associated with a change of 1.0 [50]. In our pilot study, people with COPD reported a mean improvement of 0.75 in mean score of the CRQ-F Scale after CBT-I, suggesting a clinically meaningful improvement in symptoms. Validity and reliability of the CRQ-F was demonstrated in people with COPD [49, 51, 52]. The Cronbach’s alpha for the CRQ-F in our pilot study of people with COPD and insomnia was 0.91. We measure the general feeling of fatigue using the PROMIS Fatigue CAT version described above.

#### Other control variables

We collect demographic information using a questionnaire at the screening visit. We also measure dyspnea using the CRQ Dyspnea Scale (CRQ-D). Participants rate the dyspnea they experience during selected activities that they perform on a regular basis. It is a five-item scale with a potential range from 1 = extremely short of breath to 7 = not at all short of breath. Reliability and validity were reported [53].

### Process measures

#### Health information

A clinical questionnaire is administered at baseline to characterize the sample in terms of demographics and health history. The clinical questionnaire incorporates the ATS-DLD Respiratory Questionnaire that measures dyspnea [54], the Functional Comorbidity Index [55, 56], indicators of socioeconomic status [57], and questions related to smoking status, current medications and treatment. It also includes the modified Insomnia Interview Schedule [29], which is used to explore the nature of each subject’s sleep complaint.

#### Clinical assessment

The Health Report is used to monitor health status during the intervention and the follow-up period. We monitor COPD exacerbations and the use of corticosteroids, using procedures established for a previous study (NR08037). The Health Report Form is a two-page form that is easy to complete in 1 min or less. It includes the Epworth Sleepiness Scale [58] (ESS), which is used to assess daytime sleepiness in the CBT-I group during the intervention period. These data may be tested as covariates in the analyses.

#### Daytime functioning

We will objectively measure physical activity using the Actiwatch-2® described earlier. The accelerometer at the wrist reflects whole body activity. Successful use of wrist actigraphy to quantify physical activity was reported in recent studies involving subjects with chronic illness [59, 60]. We subjectively measure daytime functioning using the PROMIS Physical Function Scale CAT version described above.

#### Treatment exposure (dose)

We examine adherence to COPD-ED and AC by keeping attendance records and CBT-I by attendance and examining sleep diary and actigraphy data. As part of each CBT-I session, sleep data are reviewed, and problems with adhering to the plan are discussed. Phone calls for assistance or to answer questions are documented.

#### Power analysis

Power analysis for aim 1 is based on main effect differences at the end of the intervention. We estimated the per cell sample size assuming a moderate effect size (Cohen’s *d* = 0.5), alpha = 0.05, and power = 0.80 using the fpower SAS macro available at http://www.datavis.ca/sasmac/fpower.html. Based on combined data from our pilot study measures, the minimum detectable difference (MDD) for the treatment main effects will be clinically meaningful. For example, we will be able to detect a 2.3-point difference in insomnia severity between those receiving COPD-ED versus those not, averaged across the other treatment conditions. The MDD for other outcomes includes Actiwatch® sleep efficiency = 6.72, CRQ-F = 0.51, and the Profile of Moods States Fatigue subscale (POMS-F) = 2.76. Between-group effect size estimates from our pilot study ranged from 0.35 for sleep efficiency to 1.03 for insomnia severity, comparing the proposed treatment components. Path models using 7–14 parameters can be estimated according to the sample size convention of 10 to 20 cases per parameter [61], thus, our simple path models can be estimated. We further conservatively estimate an attrition rate of 15 % by the end of the study. Therefore, we will enroll a total of 166 and retain 140 (35 per group) subjects in the proposed study.

#### Recruitment

The majority of subjects will be recruited through the University of Illinois at Chicago Medical Center (UICMC) and the Hines Veterans Administration (VA) Medical Centers; campus, newspaper and radio advertisements, social networking and community outreach. We contact local pulmonologists to recruit subjects through their medical practices. We also use online recruitment strategies such as Research Match. There is a focused recruitment for minorities by placing advertisements in newspapers that serve predominantly the minority communities. Similar techniques are used for women, placing advertisements in publications predominantly read by women. We employ a number of retention strategies that were successfully implemented in our pilot study.

Subjects are initially contacted by mail or advertisement, which will provide a brief description of the study. Potential subjects are asked to return a “Permission to Contact” form with their phone number so the researcher can contact them about the study. The researcher explains the study and answers questions over the phone. If the caller is interested in pursuing the study further, a preliminary health screening is conducted over the phone to determine their potential eligibility for participation. An information brochure is mailed to the home when callers want time to think before making a decision. They contact us again if they decide to pursue being in the study.

## Methods: assignment of interventions

A randomization schedule was developed using Excel whereby the four treatment groups were randomly ordered in blocks of four and sequentially assigned through REDCap according to stratum. We have four strata defined by sex and COPD severity (two levels); this process is managed separately by site. The complexity of the design (four treatment arms × four strata × two sites) is assumed to reduce predictability for the sequence of group assignment for any given participant. Non-blinded study staff generate allocation by REDCap and assign participants to interventions. Personnel administering and scoring the PFTs, actigraphy and posttest/follow-up sleep diaries are blinded to group assignment.

## Methods: data collection, data management and analysis

### Data collection

Screening procedures include an initial telephone screening to determine eligibility. Potential subjects are then scheduled for their first screening visit, which takes place at the CNSHR. At that visit, after informed consent is obtained, PFTs are performed, followed by questionnaires. Reliability and validity of pulmonary function testing have been previously demonstrated [62, 63]. The Hospital Anxiety and Depression Scale (HADS) [64] is administered to screen for major depressive symptoms. The HADS was designed for outpatients with medical illnesses, and it has been widely used in people with COPD [65–67]. Reliability and validity were demonstrated [68]. A cutoff score of 11 on the HADS depression subscale is used for depression [64]. A clinical questionnaire is completed at this visit to characterize the sample in terms of demographics and health history. Blood specimens are sent to Quest Diagnostics® for measurement of C-reactive protein and ferritin level. These tests are done in order to rule out anemia and to adequately describe the sample and may be used as covariates in the analyses.

The screening includes a one-night sleep study at home or at the UIC Sleep Science Center or the Hines VA Sleep Laboratory. The sleep study is used to screen potential subjects for sleep apnea, and low SaO_2_ during the night. Objective evidence of any primary sleep disorder according to standard clinical criteria is used to exclude subjects. The remaining subjects receive instructions for continued actigraphy monitoring by wearing an actigraph on their non-dominant wrist at home for a total of 1 week. They are instructed on how to complete the daily sleep diary.

Excluding the screening, participants have a total of eight or nine laboratory visits (six intervention, pretest, posttest and follow-up). The 3-month follow-up is completed at home or in the laboratory. After the 1-week at-home monitoring period, they are scheduled for the baseline (pretest) visit at either the UIC CON Center for Narcolepsy, Sleep and Health Research (CNSHR) or the Hines VA. Upon completion of the baseline testing, subjects are randomly assigned to group. Subjects recruited from the Hines VA attend the six intervention visits there. Subjects recruited from outside of the VA attend the six intervention visits at the UIC CNSHR. For the 3-month follow-up, subjects either come to UIC or Hines VA for a final visit or they are mailed a packet containing an actigraph, questionnaires and a self-addressed, stamped return envelope. We track attendance using attendance records and the sleep diary. All tests are performed in the same order for each subject. Tests and questionnaires are administered in a private, comfortable room. We make every attempt to minimize selection and performance bias, attrition and missing data. Selection bias is minimized by use of the randomized controlled trial design. Staff performing data analysis are blinded to group assignment. We use strategies to reduce attrition such as good communication between research staff and participants, minimizing participant burden by the use of PROMIS CAT instruments, and monetary incentives. When subjects do not complete the study, we document the reasons data are missing in order to evaluate the missing data using justified assumptions.

#### Enactment and fidelity

The clinician interventionalists are trained in CBT-I, COPD-ED, CBT-I + COPD-ED, and the AC education topics by experts. They observe several sessions being conducted by a clinician experienced in the interventions and practice the interventions under supervision. They use a manual developed during the pilot research to guide the sessions. In order to assure high-quality sessions, samples are reviewed and scored for adherence to the protocol. Subject adherence is also tracked. Adherence to stimulus control and sleep restriction is assessed at each session using the sleep diary and/or actigraph. Evaluation of adherence to stimulus control is assessed by examining responses to a sleep diary question that asks how the participant managed periods awake during the night. Evaluation of adherence to sleep restriction is assessed by comparing the recommended time in bed to the reported time in bed.

### Data management

Data are entered onto a REDCap application and stored on a secure server with daily back-ups. The raw data are stored in locked file cabinets that are housed in our research laboratory protected with a security lock. Only the immediate research staff (PI, key personnel) have access to these data files. The computerized data files are password-protected and available only to the immediate research staff (PI, key personnel). Identifiable elements are needed during the study, for instance in order to contact subjects for data collection appointments. Data are coded so that subject identifiers are on data sheets. All data are checked for completeness and validity. The quality of the data is monitored at least annually by the principal investigator (PI) assisted by the research specialists.

### Statistical analysis

#### Test of specific hypotheses

Descriptive statistics (means, standard deviation, frequency) and plots are used to screen the data prior to our main analyses. Necessary transformation and imputations are conducted based on the raw data distribution. Baseline adjustment for covariates (e.g., age, COPD severity (PFT), and gender) will be incorporated into the main effect analyses to reduce error variance and improve statistical power [69]. Data analyses is performed using SAS 9.3 statistical software (SAS Inc., Cary, NC, USA) and Mplus 6.0 (Muthén & Muthén, Los Angeles, CA, USA). All tests will be two-sided and an error rate of *α* <0.05 will be considered statistically significant. The test of each specific aim is described below, and all statistical analysis will employ an intent-to-treat approach. A fully specified statistical analysis plan will be written before unmasking.

Aim 1: to determine the efficacy of individual treatment components, CBT-I and COPD-ED, on insomnia and fatigue. In this 2 × 2 design, participants are randomized into four groups created by crossing the two factors, CBT-I versus AC1 and COPD-ED versus AC2 (see Table 3). We hypothesize that both components will improve insomnia and decrease fatigue following the six-session program, and that participants will maintain these gains at 3 months post intervention. Our factorial design permits the test of an interaction between the two treatment components, and we will assess for the presence of a strong interaction effect. However, we do not anticipate a strong synergy of the treatments since our pilot work showed that both had within-group improvement, and the hypothesized mechanisms for change differ. If the interaction is negligible, we will proceed with the interpretation of averaged main effects as shown in Table 3. The statistical tests for both components will be more robust than tests against a simple control condition because they are averaged across the levels of the other treatment. That is, the effect of each component will be tested controlling for the effect of the other. This is an efficient design because all subjects will be used to test both components. Finally, it may be more acceptable to participants since all receive supportive contact and only one out of four conditions does not receive a component hypothesized as an active treatment for insomnia in COPD patients. We will employ mixed-effects models using insomnia and fatigue as time-varying dependent variables and treatment groups as fixed effects. Demographic and clinical characteristics will be entered as time-invariant covariates if baseline group differences are observed despite randomization. Individuals’ baseline sleep measures and change over time (i.e., intercept and slope) will be modeled as random effects. The null hypothesis will be rejected if a significant treatment × time interaction is observed. For example, a significant CBT-I × time effect in the hypothesized direction would mean that participants receiving CBT-I improved more than those who did not, averaged across the other conditions. The mixed models will be run using PROC MIXED SAS 9.3 (SAS Inc., Cary, NC, USA) and estimated by residual maximum likelihood (REML). Covariance pattern structures (compound symmetry and unstructured) will also be examined, and models will be compared using likelihood ratio tests or Akaike’s Information Criteria (AIC) which is a function of the log likelihood and can be compared across models.

**Table 3:** Study design

Study design	CBT-I	AC1	Main effect COPD-ED
COPD-ED	35	35	70
AC2	35	35	70
Main effect CBT-I	70	70	

*AC1* attention control 1, *AC2* attention control 2, *CBT-I* cognitive behavioral therapy for insomnia, *COPD-ED* COPD education

Aim 2: to define the mechanistic contributors to the outcomes after CBT-I and COPD-ED. We hypothesize that CBT-I and COPD-ED components impact insomnia and fatigue through complementary mechanisms. We have previously shown that CBT-I was related to beliefs about sleep, sleep habits, self-efficacy for sleep, and that COPD-ED was related to management of COPD and emotional arousal, thus making them both appropriate candidates for an insomnia treatment approach for COPD patients. We will employ path analysis to test the conceptual model illustrated in Fig. 1 [70]. The model will test whether each treatment component is related to change in the hypothesized mediator, and whether that change is associated with improvement in the outcome. Direct and indirect treatment effects will be estimated. We will conduct path analyses as follows: (1) specification, (2) identification, (3) estimation, (4) testing of fit, and (5) respecification [71]. Potential moderators suggested by our previous work include gender, initial insomnia and fatigue severity, and inflammation and will be tested as interactions of the effect of each treatment component on the outcomes. Variables found to be a significant moderator of treatment will be tested further to determine if the effect is due to a moderated impact on significant mediator variables. Path models assume that variables used to describe relationships are manifest variables and measured without error. While we recognize that it would be preferable to estimate a measurement model, the feasible sample size for this study precludes more complex modeling. Thus, we acknowledge this limitation and will restrict path analysis to reliably measured variables, that is, with internal consistency of 0.80 or higher. Mplus (version 6) will be used to estimate the path models. The root-mean-square error of approximation (RMSEA) and Bentler’s Comparative Fit Index (CFI) will be reviewed to assess model fit. An adequate fit of the data to the model is indicated by a RMSEA value less than 0.08 and a CFI greater than 0.90.

#### Missing data

For missing data, we will determine whether missing data are MCAR (missing completely at random), MAR (missing at random), or NMAR (not missing at random). If MCAR or MAR, the standard multivariate computations will not likely result in biased standard error estimates, and full information maximum likelihood (FIML) estimation will be used. If NMAR, we will use the “pattern mixture” approach to compute a “weighted average” of the parameters associated with the missing data to estimate treatment effects.

## Ethics and dissemination

### Ethics

The study protocol is approved by the Institutional Review Board Office for the Protection of Research Subjects of the University of Illinois at Chicago and the Institutional Review Board of the Edward Hines, Jr. VA Hospital. The informed consent process begins when potential subjects are contacted. The researcher explains the study over the telephone. During the telephone consent, research staff explain the purpose of the study, study procedures, benefits, risks, confidentiality, and research subject’s rights. For potential subjects who want to come in for a screening appointment in the CNSHR, the informed consent process continues with a face-to-face explanation and discussion of the study. After all questions have been answered and the subject verbally agrees to participate, the subject signs the written informed consent and a copy of the document is provided to the subject. All staff attend the UIC IRB training program and continuing IRB education programs.

Strict procedures are in place to minimize the risk of breach of confidentiality. All subjects are assigned a code. The master list of the subject’s name and the linked code is kept in a password-protected computer in the PI’s office. All information provided by subjects is kept strictly confidential and is not be reported on an individual basis. None of the information provided by subjects becomes part of the medical record. Hard copy data are stored in a locked office, and electronic data are stored on a password-protected computer. Hard copy data and electronic data are coded, with the master list kept separately in a secure file in the PI’s office. A Health Insurance Portability and Accountability Act (HIPAA) consent form was developed for this study to use/disclose protected health information. Subjects are asked to sign the HIPAA consent form and those who refuse to agree to the HIPAA consent are not able to participate in this study.

### Dissemination

Investigators will communicate trial results to the public and healthcare professionals through publications and presentations. The final report will follow the main Consolidated Standards of Reporting Trials (CONSORT) 2010 guideline; as well as their extension to non-pharmacological interventions and to PRO outcomes.

## Discussion

### Expected outcomes and future directions

The results of this study are likely to lead to new approaches for preventive and therapeutic interventions for insomnia and fatigue. Components of therapy found to be important for positive outcomes can be included in new therapies. It is also expected that what we learn will help determine the people who are most likely to benefit from therapy. Our results will be useful in the treatment and prevention of insomnia and fatigue in people with COPD. This will lead to longer, higher-quality and more productive lives for people with COPD and reduced societal cost due to the effects of insomnia. Depending on the specific results of this study, future directions will involve developing improved, efficient and accessible insomnia and fatigue management strategies and therapies that will optimize health in patients with coexisting insomnia and COPD.

### Trial status

This study is ongoing. We are currently recruiting and enrolling subjects at the University of Illinois at Chicago and the Edward Hines, Jr. Veterans Administration.

## Abbreviations

AC, Attention Control; COPD, chronic obstructive pulmonary disease; CBTI,cognitive behavioral therapy for insomnia; COPD-ED, COPD education;COPD SES, self-efficacy for COPD; CRQ, Chronic Respiratory DiseaseQuestionnaire; DBAS, Dysfunctional Beliefs and Attitudes about Sleep;ESS, Epworth Sleepiness Scale; FIML, full information maximum likelihoodestimation; HADS, Hospital Anxiety and Depression Scale; MCAR, missingcompletely at random; MAR, missing at random; NA, number of awakeningsafter sleep onset; NMAR, not missing at random; PFT, pulmonary functiontest; PROMIS, Patient Reported Outcomes Measurement Information System;RMSEA, root mean square error of approximation; SE, sleep efficiency;SES, self-efficacy about sleep; SII, Sleep Impairment Index; SL, sleep onsetlatency; TST, total sleep time; VA, Veterans Administration; WASO, wake aftersleep onset.
